# Supplementary material for: Are carbon emissions trading and green financial instruments synergistic? -Comprehensive quantitative research based on content analysis
Source: PLoS One. 2024 Mar 7;19(3):e0298601. doi: 10.1371/journal.pone.0298601 (PMC10919601; doi:10.1371/journal.pone.0298601)
Supplement: S1 Data — (ZIP) [file pone.0298601.s001.zip › Data/地级市Z_F工作报告.zip!/║╙─╧/░▓╤⌠╩╨/└┤╫╘╣1⁄2╓┌║┼í╛┬φ┐╦╩2╛▌═°í┐_2009.docx]

各位代表：

    现在，我代表市人民政府，向大会报告政府工作，请予审议，并请各位政协委员提出意见。

    一、过去五年工作回顾

    2004年以来，市政府在省委、省政府和市委的正确领导下，以邓小平理论和“三个代表”重要思想为指导，深入贯彻落实科学发展观，开拓进取，扎实工作，圆满完成了市十一届人大一次会议确定的各项目标任务。

    ——经济实力快速提升。五年来，紧紧围绕“一强五前”的目标，大力实施“四路并进”，经济社会较快发展。全市生产总值由2003年的353.7 亿元增加到2008年的1036亿元，年均增长15.5%，高于全国、全省年均增速。人均生产总值达到19924元，是2003年的3倍，年均增长15.1%；限额以上工业增加值达到501.3亿元，是2003年的3.9 倍，年均增长22.7%；财政一般预算收入达到50.1亿元、支出达到95.2亿元，分别是2003年的2.8 倍和2.7倍，年均分别增长23%和22%；全社会消费品零售总额达到245.8亿元，是2003年的2.2倍，年均增长17.4%。

    ——产业结构得到调整。五年来，积极调整产业结构，产业竞争力进一步提升。工业实力增强。强力推进“工业振兴工程”，工业增加值占生产总值的比重由2003年的45.1%提高到2008年的57%。实施千万元以上的项目259个，累计投资219亿元。安钢120吨转炉及炉卷轧机、鑫盛数控机床、凯瑞数码、安烟技改工程等99个项目建成投产。沙钢永兴中厚板轧机、林丰铝电25万吨高性能铝合金、中升3000吨多晶硅、新能光伏40兆瓦薄膜太阳能电池等项目进展顺利。积极推动冶金建材、煤化工、装备制造、电子信息、食品加工、纺织等优势产业和高新技术产业发展，培育壮大百户重点企业和50户高成长性企业。全市销售收入超亿元的企业达到190 家，超10亿元的达到29家，超50亿元的达到3 家，13家企业入围省百强企业。2008年高技术产业增加值14.9亿元，比上年增长24.7%。关停小钢铁、小水泥等污染企业346家，在全省率先完成小火电机组关停任务，全部淘汰18家23条水泥机立窑生产线。2008年单位GDP能耗较上年下降6.78%，超额完成省政府确定的污染减排目标任务。第三产业发展势头良好。2008年增加值达到245.7亿元，是2003年的2.2 倍。编制了文化、旅游、现代物流、商业网点等产业发展规划，金融、房地产等新兴服务业加快发展。五年累计接待国内外游客5107万人次，旅游总收入183.6 亿元。丹尼斯、大华、沃尔玛等一批商贸物流集团落户我市，“八挂来网”升格为省交通物流信息系统，被列为全国交通行业节能减排重点示范项目。

    ——“三农”工作明显加强。五年来，大力实施“四大兴农计划、三大惠民工程、十大农业产业链、十大增效技术和林业生态市建设”，全市粮食总产量累计达到1440万吨，连续五年丰产丰收。免除农业税，累计落实粮食直补、农机补贴等各类补贴4.8亿元。现代农业快速发展。新建农业龙头重点企业118家、大型农产品加工基地区4个，新建百亩园、千亩方、万亩区143个，新增瓜果菌菜等高效农业面积100万亩。新建畜禽养殖密集区299个、大中型养殖企业225家。粮食、畜牧等十大农业产业链进一步拉长，带动农产品生产基地354万亩。林业生态市建设进展顺利，新增造林面积110万亩，完成了1500个村围村林和3500公里通道绿化任务。全市有效灌溉面积达到440万亩，红旗渠、漳南灌区技改和南谷洞、石门、彰武、南海、汤河等大中型水库除险加固工程顺利实施。新农村建设扎实推进。“十大标兵村”和130个试点村建设成效显著。乡村清洁工程顺利实施，897个村建立了垃圾处理站，配备了清洁卫生员。新增农村沼气用户19万户，解决110万人安全饮水问题，完成农村劳动力转移培训 57万余人。新建、改造农村公路3035公里。完成213个贫困村整村推进任务，解决了19.2万农村贫困人口的脱贫和温饱问题。

    ——城乡面貌显著改观。五年来，积极优化投资结构，加大了城乡建设力度。重点项目积极推进。累计完成全社会固定资产投资1693.7亿元，建成了近400个省、市重点项目。安林高速、安南高速、濮鹤高速内黄段、新长高速滑县段、大广高速滑县段建成通车，安姚公路、安新高速扩宽改造、南水北调安阳段、马家岩水库等项目顺利实施。城市建设和管理不断加强。城市总体规划修编纲要通过建设部技术认定，土地利用总体规划通过省验收。开发区、安东新区建设步伐加快，旧城及街景改造进展顺利。水、电、气、暖等基础设施功能不断完善。大唐安阳电厂2×30万千瓦热电联产机组建成使用。新增城市道路32公里、经济适用住房85.1万平方米、城市公共绿地712万平方米、城市集中供热348万平方米、天然气用户5.2万户，治理背街小巷557条。东北南外环、中华路开发区段、华祥立交桥等建成通车。实施精细化管理，我市荣获“中国城市管理进步奖”。城镇化水平进一步提高。城镇化率达到37.3%，比2003年提高6.8个百分点。“一申四创”和“新四创”工作扎实推进。殷墟列入世界文化遗产保护名录，成功创建中国优秀旅游城市、全国科技进步先进市、省园林城市和省文明城市，创建国家卫生城市、国家园林城市工作正在积极推进。

    ——改革开放不断加快。五年来，积极推进体制机制创新，加快发展开放型经济，发展活力进一步增强。各项改革深入推进。累计完成国有企业改革112家。基本完成国有工业企业产权多元化改革任务，国有资产监管体制初步建立。华强、沙钢、永煤、首创、保利、香港华润、河南中孚等公司通过增资扩股等形式分别与我市的企业实现战略重组。农村综合改革成效显著。粮食流通体制改革顺利完成。市商业银行、邮政储蓄银行挂牌运行。农业银行股改确权工作积极推进。市信用担保公司增资扩股任务完成。九天化工、神州万象和瑞能清洁能源在境外成功上市。行政管理体制改革和分配制度改革稳步推进。科技、教育、文化、卫生等领域改革进一步深化。非公有制经济快速发展。增加值由2003年的158亿元增加到518亿元，占生产总值的比重达到50%，提高6个百分点。对外开放水平进一步提高。开放型经济成效显著，对外交流与合作空前活跃。五年实际利用外资3.1亿美元，年均增长47.6%，是上一个五年的6.6 倍；外贸出口28.5亿美元，年均增长41.9%，是上一个五年的4.6倍。县域经济逐步壮大。县域生产总值734.6亿元，占全市生产总值的比重达到70.9%，比2003年提高10.2个百分点。县域财政一般预算收入19.8亿元，年均增长21.6%。

    ——人民群众得到较多实惠。五年来，坚持以人为本，着力改善民生，力求让人民群众享受到更多发展成果。五年累计解决事关群众切身利益的实事近200件。城乡居民收入稳步增加。2008年城镇居民人均可支配收入13637元，农民人均纯收入5190元，分别是2003年的1.9倍和2.1倍。城乡居民储蓄存款由2003年的319 亿元增加到526.5亿元，增长65%。就业工作成效显著。五年新增城镇就业54万人，先后有9.34万名下岗失业人员再就业，发放小额担保贷款3.43亿元，扶持8600人成功创业。在全省率先基本消除城镇零就业家庭，开展了农村零转移就业家庭帮扶活动。社会保障工作进一步加强。“两个确保”持续巩固，并轨工作顺利完成。企业养老保险实现省级统筹。工伤保险扩面工作进展顺利。“双困”家庭住房保障实现应保尽保。在全省率先建立城市居民最低生活保障制度。农村低保覆盖人口13.8万人。新建、改扩建农村敬老院200所，集中供养率达到45%。城乡医疗救助工作全面铺开，慈善事业快速发展。新型农村合作医疗实现全覆盖，五年投入6亿元补贴农民医疗费。对全市乡镇卫生院、疾病预防控制机构、城市社区卫生服务中心进行了建设和改造，初步完善了城乡居民基本医疗和公共卫生服务体系。

    ——社会事业全面进步。五年来，不断加大社会事业投入，实现了经济与社会协调发展。科技进步对经济增长的贡献率达到48%，比2003年提高6个百分点。“两基”工作顺利通过国家验收，新增高中阶段学校24所，安阳工学院实现“专升本”，安阳师院通过教育部本科教学水平评估。落实5.7亿元资金，对农村义务教育阶段学生实行“两免一补”，惠及学生330万人次。全部免除了城市义务教育阶段学生杂费。我市被评为“全国未成年人思想道德建设工作先进城市”。市图书馆、博物馆建成使用，中国文字博物馆建设进展顺利，推出了《大爱无言》、《红旗渠》、《家在洹上》等一批文艺精品。深入开展群众性体育活动，建成中国体育彩票安阳全民健身活动中心和446处遍布城乡社区的健身园，成功举办市八届、九届运动会，圆满完成奥运火炬安阳传递活动。大力培养和引进人力资源，人才工作得到加强。人口和计划生育工作成效显著，连续六年受到省委、省政府表彰。圆满完成了第一次全国经济普查任务。第二届《安阳市志》顺利出版。

    精神文明和民主法制建设进一步加强。社会主义荣辱观教育和精神文明创建活动深入开展，涌现出了谢延信、周国允、吴新芬、王生英、陈建星等一批先进典型人物。坚持依法治市，严格依法行政，自觉接受人大法律监督和政协民主监督，五年累计办理人大代表议案18件、建议1541件和政协委员提案3010件。安全生产工作成绩显著，连续五年被评为完成安全目标优秀市。加强社会治安防控体系建设，依法做好信访工作，建立健全应急机制，社会大局保持稳定。农村基层民主政治建设稳步推进，完成了第五次、第六次村委会换届选举工作。“双拥”共建工作深入开展，先后4次被命名为“全国双拥模范城”。全市国防后备力量建设与经济融合式发展取得了重要进展，应对多种安全威胁能力明显提高。廉政建设和反腐败斗争取得明显成效，工作机制体制逐步理顺，经济发展环境进一步改善。不断加强老区建设，依法保护妇女儿童合法权益，老龄工作和关心下一代工作健康发展。民政、统计、审计、档案管理、经济研究、外事侨务、民族宗教、红十字等工作也都取得了新成绩。

    刚刚过去的2008年，是经济社会发展遇到困难最多的一年，也是取得成绩较大的一年。一年来，面对复杂多变的经济形势，我市迎难而上，积极应对，确立了“扭住工业，抓好农业，加快发展第三产业”的发展思路，规划建设“三大工业功能区”，发展壮大“七大主导产业”，制订了“城市建设三年计划”，实施了“四百工程”等一系列保增长、促发展的政策措施，经济社会保持了平稳较快发展。全市生产总值完成1036亿元，比上年增加228亿元，增长13.1%，财政收入增长17.8%，城镇固定资产投资增长33.1%，社会消费品零售总额增长23.8%，进出口总额增长92.8%，城镇居民人均可支配收入实际增长8%，农民人均纯收入实际增长7.6%，科技、教育、文化、卫生、体育等社会事业全面发展，为圆满完成本届政府的目标任务提供了坚实保证，也使我市的发展迈上了新起点，步入了科学发展、和谐发展、跨越发展的新阶段。

    各位代表！

    这些成绩的取得，是市委正确领导的结果，是市人大、市政协大力支持的结果，是全市人民齐心协力、拼搏奉献的结果。五年来，勤劳智慧的安阳人民，用激情和汗水谱写了不畏艰难、开拓进取的奋斗篇章，谱写了同心同德、和衷共济的团结篇章。在此，我代表市政府，向奋斗在全市各条战线上的广大工人、农民、干部、知识分子、解放军指战员、武警官兵、政法干警以及各界人士，致以崇高的敬意！向所有关心、支持我市现代化建设的港澳台同胞、海外侨胞、国际友人，表示衷心的感谢！

    各位代表！

    五年的不懈努力，创造了改革发展的丰硕成果。五年的生动实践，积累了许多宝贵的经验。我们深刻体会到，要把安阳的各项事业不断向前推进，必须始终坚持解放思想、实事求是、与时俱进，把中央和省委的精神与安阳实际紧密结合起来，深化市情认识，适应形势变化，抢抓发展机遇，积极探索体现时代特征、具有安阳特色的发展路子；必须始终坚持发展这个第一要务，紧紧扭住经济建设不放松，紧紧抓住项目带动不动摇，重视做好打基础、增后劲、利长远的工作，大力培育新的经济增长点，不断提高发展质量和效益；必须始终坚持改革开放，以深化改革激发发展活力，以扩大开放拓展发展空间，以优化环境聚集发展资源，不断为经济社会发展注入强大动力；必须始终坚持以人为本，更加注重发展成果的普惠性，大力推进社会建设和民生改善，着力解决广大群众最关心、最直接、最现实的问题；必须始终坚持科学行政、民主行政、依法行政，切实加强政府自身建设，大力推进政府管理创新，提高领导科学发展的水平和驾驭复杂局面的能力。

     在肯定成绩的同时，我们也清醒地看到，政府的工作与全市人民的期待还有一定差距，经济社会发展还存在一些矛盾和问题。一是受国际金融危机影响，我市经济受到巨大冲击，企业经营困难，市场需求不足，产品价格下降，传统产业遇到了前所未有的困难和挑战。二是经济发展方式没有实现根本性转变，产业层次低，经济发展高度依赖投资、过度依赖资源的问题没有根本解决。三是县域经济发展不平衡，县与县之间在经济总量、财政收入等方面差距有继续拉大的趋势。四是就业形势依然严峻，社会保障压力较大，教育、医疗、住房等群众关心的热点难点问题还没有根本解决，困难群体的救助帮扶机制还需要进一步完善。五是有的地方和部门抓改革、抓发展的紧迫感不强，主动性不够，部分机关工作人员办事效率低，缺乏主动服务、超前服务的意识等。对此，我们一定要高度重视，在今后的工作中采取有效措施，认真加以解决。

    二、今后五年总体要求和奋斗目标

    未来五年，既是我市加快建设豫北区域性中心强市、全面建设小康社会极其关键的五年，也是由“十一五”跨入“十二五”非常重要的五年，既是充满机遇、面临挑战的五年，也将是励精图治、大有作为的五年。30年改革开放的伟大成果为我市加快发展奠定了物质基础，政通人和的社会环境为我们提供了干事创业的良好氛围。我们一定要牢牢抓住这一战略机遇期，不断采取各项工作的新举措，奋力实现经济社会发展的新跨越。

    今后五年政府工作的总体要求是：以邓小平理论和“三个代表”重要思想为指导，深入贯彻落实科学发展观，紧紧围绕建设豫北区域性中心强市目标，扭住工业，抓好农业，加快发展第三产业，大力实施项目带动战略，着力构建“一个载体、三个体系”，坚定不移地深化改革和扩大开放，持之以恒地改善民生和加强社会建设，努力促进经济社会又好又快发展，让河南的“北大门”更加亮堂、更加光彩，为中原崛起作出应有的贡献。

    今后五年的奋斗目标是：

    ——综合实力明显增强。在优化结构、提高效益、降低消耗、保护环境的基础上，全市生产总值突破2000亿元，年均增长9%以上，人均生产总值达到38000元。工业增加值达到1200亿元，年均增长10%以上。财政一般预算收入高于生产总值增长。

    ——社会发展明显加快。城乡居民收入不断增加，城镇居民人均可支配收入、农民人均纯收入分别达到19000元和6900元。城乡义务教育均衡发展，基本医疗卫生服务明显改善，劳动就业更加充分，覆盖城乡居民的社保体系初步建立。城镇化率达到44%以上。

    ——生态环境明显改善。单位生产总值能耗和主要污染物排放总量完成省定目标，林木覆盖率达到30%以上。

    各位代表！

    伴随着我国改革开放的伟大历史进程，我市已经进入了一个全新的发展阶段，踏上了向经济强市和文化强市跨越的新征程。我们相信，再经过五年或更长时间的努力，一个经济日益发达、文化日益繁荣、社会日益和谐、生态日益文明的新安阳，必将在豫北大地率先崛起！一个更有实力、更具活力、更富魅力的新安阳，必将焕发出更加璀璨夺目的光彩！

    三、2009年工作安排

    2009年是新世纪以来经济发展最为困难的一年，也是蕴涵机遇最大的一年。国际金融危机的影响还会进一步加深，发展面临的形势异常复杂、异常严峻。因此，全市上下要坚定信心，知难而进，从变化的形势中捕捉和把握发展机遇，认真贯彻落实中央和省委“扩内需、保增长，调结构、上水平，抓改革、增活力，重民生、促和谐”的决策部署，保持经济社会平稳较快发展，保持跨越式发展的良好势头，为更长时期、更高水平的发展奠定基础。

    今年经济社会发展的主要目标是：生产总值增长10%；地方财政一般预算收入增长10%；限额以上工业增加值增长13%；全社会固定资产投资增长20%；社会消费品零售总额增长14%；实际利用外资增长15%；居民消费价格指数涨幅控制在4%左右；城镇居民人均可支配收入增长8%，农民人均纯收入增长7%；人口自然增长率控制在6.3‰以内；城镇登记失业率控制在4.6%以内，城镇新增就业人数6.5万人；单位生产总值能耗下降6.02%，化学需氧量、二氧化硫排放量完成省定目标。

    在实现上述目标的同时，市委、市政府今年还要全力为全市人民办好十个方面实事。这十个方面实事会上已印发，请一并审议。

    围绕上述目标，今年将重点抓好以下八个方面的工作：

    （一）全力促进经济平稳较快发展。进一步增强经济运行的预见性、科学性和有效性，采取更加有力的政策措施，促进经济平稳较快发展。

    深入实施项目带动战略。进一步扩大投资规模，着力抓好重点项目建设，加快推进“8155”投资计划。在农林水利、交通、能源、城镇建设、自主创新、产业升级、节能减排、社会事业八大领域，建设100个以上超亿元的重大项目，带动全社会新开工项目总投资规模超500亿元，力争全市今年完成城镇固定资产投资超500亿元。交通方面，重点抓好安姚公路和安新高速公路扩建、石武铁路客运专线、山西中南部铁路对外通道、林长高速公路、鹤辉高速公路等线路安阳段开工建设，努力打造国家级交通枢纽城市。能源方面，重点抓好鑫龙煤业红岭煤矿技改、超越实业王家岭新井、林州2×30万千瓦热电联产机组等项目建设；确保安阳西500千伏输变电、榆林至济南天然气管线安阳段等项目开工；适时启动大唐林州、华润安阳百万千瓦发电机组等项目前期工作。农田水利方面，大力推进南水北调中线安阳段建设和红旗渠、漳南灌区等节水技改工程，加快建设弓上、小南海、双泉等水库除险加固工程。民生方面，加快建设宗村污水处理厂、热力公司管网二期扩建等项目，推进会展体育中心、体育训练中心、老年活动中心、儿童福利院等项目建设。支持具有国家自主知识产权的第三代移动通信TD建设。

    着力扩大消费需求。把调整收入分配、发展服务业与扩大消费需求结合起来，大力开拓消费市场。提高居民消费能力。改革事业单位工资制度，健全企业最低工资制度和农民工工资支付保障机制。提高城乡居民最低生活保障水平。提高优抚对象、建国前老党员抚恤和生活补助标准。提高企业退休人员基本养老金水平，人均月增加100元以上。提高农村“五保”集中供养标准，将年供养标准由1400元提高到1600元。增加对高等院校家庭困难学生的生活补助。积极培育新的消费热点。引导房地产企业建设中低价位的普通商品房，鼓励大型房地产企业连片开发。加快发展住宿餐饮、社区商业、物业、家政、休闲、文化等服务性消费。继续优化消费环境。全面清理消费领域相关规费。继续实施“万村千乡市场工程”，建设300多个农村便民超市，增加“家电下乡”补贴品种。

全面落实财政金融政策。落实积极的财政政策，综合运用多种手段扩大投资，引导带动社会投资。依法加强税收征管，规范非税收入管理，优化支出结构，加快资金拨付进度，确保法定支出、工资发放、改善民生、落实配套资金、“十个方面实事”等资金需要。充分发挥金融促进经济发展的作用，鼓励金融机构在风险可控的前提下，加大信贷投入，全年新增贷款确保50亿元。加快中小企业信用担保体系建设，全面建立市、县两级中小企业信用担保体系。

    深入开展“企业服务年”活动。加强对重点行业、骨干企业和主要产品的监测分析，及时采取对策。进一步搞好经济运行调控调节，组织好煤电油气运等供需衔接，保障企业要素资源供给。落实领导分包责任制，主动帮助企业解决生产经营中的困难。落实涉企优惠政策，切实减轻企业负担。继续组织企业、项目单位开展供需对接等多种活动，形成稳定的合作关系，提高抗风险能力。优化企业发展环境，严查涉企“五霸”案件。

    （二）加快推进发展方式转变。加快结构调整和发展方式转变是应对挑战的应急之策，又是实现可持续发展的根本大计，必须因时而动，顺势而为，着力构建“一个载体、三个体系”。

    大力发展产业集聚区。把产业集聚区作为优化经济结构、转变发展方式、实现集约化发展的载体。切实做好列入省规划的九大产业集聚区建设，加快编制我市西部新型制造业、中部高新技术产业、东部农副产品加工业三大工业功能区规划，培育壮大冶金建材、煤化工、装备制造、电子信息、食品加工、纺织和新能源七大支柱产业。加快产业集聚区基础设施和结构调整项目建设，提升对城市产业、人口、环境的承载功能。推动企业向园区集中、园区向城镇集中、劳动力向城镇转移，促进经济发展与资源环境良性循环，实现产业与城市发展相互推动，逐步形成集聚促进就业、就业创造消费、消费拉动经济增长的良性发展机制。

    加快构建现代产业体系。优化要素资源配置，推行集约化发展模式，构建现代农业、工业主导产业、高新技术产业、现代服务业、基础设施和基础产业等相互支撑、互动发展的现代产业体系。继续推进“工业振兴工程”。重点抓好81个投资5000万元以上的工业项目建设。推进安钢集团冷轧薄板和中板热处理、安化集团甲醇、永兴中厚板、安锻数控锻压设备、三丰铜板带、轻机纸浆设备、林丰铝合金、益海嘉力面粉加工、滑县服装加工基地、中福陶瓷高档地板砖等项目建设。加快编制新能源产业规划，支持中升多晶硅、凤凰多晶硅、欧美亚单晶硅、新能光伏薄膜太阳能电池、安彩高科光伏玻璃、嘉能能源二甲醚等项目建设，着力打造新能源谷。加快结构调整和企业战略重组。依托骨干企业加快联合重组，组建大型钢铁集团。支持凤宝等20家无缝钢管企业整合，推进顺成、利源、豫龙、鑫磊等焦化企业以及安化集团与永煤集团战略合作，打造河南省精品钢生产基地和煤化工生产基地。大力发展服务业。充分发挥服务业引导资金作用，强力推进沃尔玛、“八挂来网”、彰德府威尼斯印象、开发区铁路专用线及物流园区建设，开工建设汤阴粮食物流中心、安阳义乌小商品城等项目。积极发展房地产业和金融、法律等现代服务业。全面整合旅游资源，加大旅游基础设施建设力度，深度开发系列旅游产品和线路，强力打造“殷墟、林虑山、周易”三大品牌，将其建设成为国家5A级旅游风景区。今年以推介“山水安阳”为重点，带动“文化安阳”、“历史安阳”共同光大，努力实现“三阳开泰”。

    加快构建现代城镇体系。全面启动“城市建设三年计划”，积极推动人口、产业和生产要素向城镇和产业带聚集。认真做好城市规划。高标准制订城市分区规划和重要部位、重要地段、重要节点控制性详细规划，编制安东新区、老城区保护和开发规划，完成“城中村”改造、街景整治等规划。加强城市建设。实施“六路三片区”建设工程，抓好光明路等6条道路和京港澳高速南北出入口、红旗渠广场3个片区建设。实施人民大道、解放大道等9条路街景整治，综合治理洹河、洪河、环城河及市内沟渠坑塘，构建城市水系。围绕创建“国家卫生城市”和“国家园林城市”目标，开展城市环境综合治理，提高城市管理水平，逐步实现城市管理网格化、信息化。加快城镇化进程。抓好林州市区、滑县、汤阴县、内黄县县城以及安阳县水冶镇和柏庄、白璧、宝莲寺等卫星镇建设，努力构建城乡协调发展、共同繁荣的城市体系。

    加快构建自主创新体系。制订实施自主创新体系规划，落实加快科技创新促进产业发展的意见，坚持以企业为主体，以应用开发研究为重点，以重大科技专项为抓手，以构建区域创新体系为基础，以创新体制机制为动力，集中科技资源，形成一批具有全省乃至全国先进水平并拥有自主知识产权的科技成果。加大全社会研发投入，大力支持大中型企业建立工程技术中心、企业技术中心等研发中心。大力发展循环经济，认真落实节能减排问责制和“一票否决”制，严把新建项目准入关，加强电力、煤炭等八大重点行业节能降耗。

    （三）切实加强“三农”工作。把解决好“三农”问题作为重中之重，围绕稳粮、增收、强基础、重民生，继续大力实施四大兴农计划、三大惠民工程、十大农业产业链开发、十大农业增效技术和林业生态市建设，扎实推进新农村建设。

    巩固提高粮食生产能力。认真落实国家粮食、农机、农资、畜牧等各项直补政策，突出抓好东部350万亩粮食生产核心区建设，全面提升我市农田抗旱除涝水平，确保粮食种植面积稳定在770万亩以上，总产稳定在330万吨左右。

    大力发展现代农业。全年培植农业产业化和农村特色经济乡（镇）30个，提升县（市）区农产品加工区的规模档次。进一步拉长粮食、棉花、畜牧、林木等十大农业产业链链条，提高终端产品水平，全面提高农产品市场竞争力。新增亩均效益3500元的高效农业面积15万亩，新建高效农业示范园区30个。加快实施“552”现代畜牧业发展计划，新增畜禽饲养密集区100个，大中型规模养殖加工企业50个。

    扎实推进新农村建设。今年市级财政安排700万元资金，加快130个试点村建设。完善扶贫开发长效机制，积极争取国家扶贫资金，完成86个贫困村整村推进和4.5万农民脱贫任务。继续推进生态家园富民沼气工程和乡村清洁工程，新增沼气用户4.5万户，新建大中型沼气池70座，完成15万人安全饮水和10万人“民办公助”自来水工程。大力推进县、乡公路建设，确保新建农村公路700公里。推进林业生态市建设，新增营造林面积45.29万亩。发展和改造村级粮油、供销超市1000个。完成198个行政村的农民体育健身工程建设任务。

    全面深化农村改革。稳定和完善农村基本经营制度，加强土地承包经营权流转管理和服务，按照依法自愿有偿的原则，允许农民以转包、出租、互换、转让、股份合作等形式流转土地承包经营权。抓好林业“四荒”拍卖造林体制改革、农村中小型水利设施体制改革、集体林业产权制度改革。改善和创新农村金融服务，推进村镇银行和小额贷款公司建设，完善支持农民小额贷款办法。加快农民专业合作组织发展，转变运作机制，激发内部活力，充分调动社会资金投向“三农”。

    （四）不断推动文化大发展、大繁荣。进一步提升文化软实力，为建设豫北区域性中心强市提供强大的思想保证、舆论支持和文化支撑。

    着力发展文化事业。抓好“五个一工程”，创作一批文化精品，繁荣发展文化事业。加快推进中国文字博物馆、殷墟大遗址公园等标志性文化工程建设，尽快建成一批布局合理、开放性好、实用性强的群众文化基础设施。推进全市博物馆、纪念馆免费开放。抓好乡（镇）综合文化站建设和文化信息资源共享工程，扩建改造11个乡（镇）综合文化站，建成2个达到国家标准的文化信息资源共享县级支中心、1549个村级基层点和280个农家书屋。继续实施农村电影放映工程，开展“千场舞台艺术送农民”活动。切实加强重点文物和非物质文化遗产保护。进一步办好节庆活动，组织策划好殷商文化旅游节、全国特种养殖种植技术成果博览会等活动，形成一批具有重大影响的文化品牌。

    加快发展文化产业。全面落实文化产业扶持政策，重点发展新闻出版、广播影视、演艺娱乐等文化产业。做好文化科技项目和文化创意项目。积极发展特色文化产业，推动安阳刺绣、汤阴剪纸、内黄农民画等特色文化产业发展。

    推进文化体制改革。积极探索、努力建设文化产业的聚集地、文化创新的示范区。加快经营性文化事业单位转企改制，完善文艺院团“一团一策”改革实施办法。深化公益性文化事业单位内部人事、收入分配和社会保障制度改革。拓宽文化产业融资渠道，鼓励非公有资本进入文化娱乐领域，培育优势民营文化企业。

    （五）深入推进改革开放。着眼于完善社会主义市场经济体制，采取有效措施，加快改革开放步伐。

    积极推进改革创新。继续深化国有企业改革，力争两年内基本完成国有企业产权制度改革任务。完善国有资产监管体制，确保国有资产保值增值。积极推进集体企业改革，推进供水、供气、供热等公用事业产业化、市场化进程。深化财税、价格体制改革，实现政府公共预算、国有资本经营预算有机衔接。实施好新修订的增值税、营业税、消费税暂行条例及燃油税费改革方案，抓好资源税等改革。

    全力提高开放水平。落实招商引资奖励政策，实施一批重大引资项目。组织高效益的专项招商引资活动，紧紧盯住世界和全国500强企业、中央企业的战略布局调整和投资意向，扎实引进战略投资者。加强区域经济合作，积极促进我市与省内外其他城市，与环渤海、珠三角、长三角地区和中原协作区建立更加密切的经济联系，实现互利共赢。下大力气调整进出口结构，促进对外贸易稳步持续增长。

    进一步发展壮大县域经济。优化县域产业布局，促进县域产业集约集群发展。统筹安排县域经济发展奖补资金，加大对县级转移支付力度，支持县域产业项目建设，力争每个县( 市)都要引进一批具有牵动力的重大项目，切实增强县域经济实力。

    大力发展非公有制经济。进一步改善环境，放宽市场准入，鼓励非公有制企业投资金融服务、基础设施、公用事业领域。认真落实促进非公有制经济发展的各项政策措施，加快建立健全非公有制经济社会化服务体系，支持非公有制企业总量上规模、结构上档次、质量上水平、管理上台阶，不断提高非公有制经济对全市经济发展的贡献率。

（六）切实加强以改善民生为重点的和谐社会建设。把民生工程作为推进公共服务均等化的重要措施，努力使公共服务向农村延伸，向基层延伸，向困难群体延伸。

    千方百计扩大就业。全面落实全民创业意见，推动以创业促就业，落实好大学生就业和农民工返乡创业扶持政策。进一步开发公益性岗位，继续延长“4050”等社会困难人员享受公益性岗位就业的各项补贴政策，动态解决“零就业家庭”就业问题。扩大实施“农村劳动力技能就业计划”、“阳光工程”和“雨露计划”。力争实现下岗失业人员再就业3万人，其中就业困难人员再就业1万人。加大农村劳动力转移力度，新增转移就业5万人，总数达到155万人，劳务输出95万人。

    着力完善社会保障体系。逐步扩大基本养老保险覆盖范围，城镇职工基本养老保险新增参保人员2.5万人。完善失业保险制度，确保失业保险金按时足额支付。全面实施被征地农民社会保障制度。尽快将关闭破产企业退休人员和困难企业职工纳入城镇职工医保。积极推进城镇居民医疗保险工作，争取在2010年前基本实现全覆盖，今年将在校大学生全部纳入城镇居民医疗保险范围。加快推行城乡居民医疗救助制度。扩大工伤保险覆盖面，力争参保人数达到32万人。加大保障性住房投资建设力度，开工建设廉租住房7.5万平方米，竣工1.5万平方米，为城市低收入家庭提供300套廉租住房。开工建设经济适用房20万平方米、竣工10万平方米，为城市低收入家庭提供1000套经济适用房。继续实施农村贫困残疾人危房改造工程，再改造100户危房。

    加快发展科技、教育、卫生、体育、计划生育等社会事业。加强科技基础条件平台建设，深入实施新农村建设科技示范、科技成果转化、知识产权优势培育等科技工程，大力发展高新技术产业，实施知识产权战略纲要，积极创建“国家知识产权试点城市”，继续争创新一轮“全国科技进步先进市”。全面实施城乡免费义务教育，农村中、小学义务教育学校生均公用经费标准分别由375元和230元提高到500元和300元，继续对农村义务教育阶段学生全部免除学杂费、免费提供教科书，补助农村寄宿制学校家庭经济困难学生生活费。继续免除城市义务教育阶段学生学杂费。加快普及高中阶段教育，大力发展职业教育，进一步完善高等教育结构，重点做好安阳职业技术学院筹建、安阳中医药学校搬迁工作。进一步完善新型农村合作医疗制度，加强新农合资金的监督管理。积极推进城市社区卫生服务网络建设，完善社区卫生服务职能。建设54个标准化村卫生室，为65个乡（镇）卫生院配备基本医疗设备，完成17个薄弱乡（镇）卫生院改造。加强体育基础设施建设，提高全民健身和竞技体育水平，认真办好滑翔伞世界杯热身赛暨全国滑翔伞优秀选手赛。完善人才政策，吸引和用好各类人才。稳定低生育水平，提高出生人口素质，综合治理出生人口性别比偏高的问题。农村部分计划生育家庭奖励扶助标准由每人每年600元提高到840元。

    认真做好安全生产、食品药品安全和社会稳定工作。深入开展安全生产隐患排查治理，抓好煤矿、交通运输、烟花爆竹等重点行业和重点企业的安全管理。深入开展整顿和规范市场秩序专项行动以及“质量和安全年”活动，加大对食品药品安全专项整治，坚决杜绝不合格产品进入市场。加强基层信访工作，畅通群众利益诉求表达渠道。大力推进法律援助工作。加强社会治安综合治理，深入开展平安创建活动，依法防范和打击违法犯罪活动，切实保障人民群众生命财产安全。

    （七）深入推进精神文明和民主法制建设。以弘扬红旗渠精神为载体，加强社会主义核心价值体系建设。深入开展学习实践科学发展观活动，提高干部群众推动科学发展的能力和水平。继续开展“知荣辱、讲正气、促和谐”主题道德实践活动，加强诚信体系建设。积极争创第二届全省文明城市，全面提升市民文明素质。加强未成年人思想道德建设，进一步净化社会文化环境。深入开展“清洁家园行动”，改善农村人居环境。积极组织开展志愿服务活动，继续推进文化科技卫生“三下乡”，推动群众性精神文明创建活动蓬勃开展。

    继续做好国防动员、人民防空工作，加强全民国防教育和民兵预备役建设，深入开展“双拥”共建活动，落实优抚安置政策，支持国防和军队建设。进一步增进民族团结，处理好宗教事务，发挥好宗教界人士和信教群众在促进经济社会发展中的积极作用。充分发挥工会、共青团、妇联等人民团体的桥梁纽带作用。深化城市社区建设，搞好农村社区建设试点。积极做好老龄和关心下一代工作。加强社会福利事业建设。进一步做好外事侨务工作。继续做好人事、民政、统计、审计、地震、档案管理、经济研究、红十字等工作。

    （八）进一步加强政府自身建设。按照建设学习型、法治型、廉洁型、服务型政府的要求，坚持不懈地推进“两转两提”，切实提高行政能力和服务水平。

    转变职能，提高政府服务质量。加快推进政企分开、政资分开、政事分开、政府与市场中介组织分开，更加注重履行社会管理和公共服务职能。积极稳妥地推进新一轮政府机构改革。深化行政审批制度改革，减少和规范行政审批事项，进一步减少行政事业性收费。完善公共财政体系，把更多财政资金投向公共服务领域。创新社会管理体制，建立健全科学有效的利益协调、社会纠纷调处和权益保障机制。认真搞好第二次经济普查等国情国力调查。重视发挥新闻舆论和社会公众的监督作用。深入推进政务公开，保证群众的知情权、参与权和监督权。

    依法行政，提高政府管理能力。深入贯彻落实《行政许可法》和《全面推进依法行政实施纲要》，严格按照法定权限和程序履行职责。认真执行人大及其常委会各项决议、决定，自觉接受人大及其常委会的法律监督和工作监督；加强与人民政协的协商，自觉接受人民政协的民主监督；广泛听取各民主党派、工商联和无党派人士的意见和建议。认真办理人大代表建议和政协委员提案。严格规范行政执法行为，确保严格执法、公正执法、文明执法。完善应急预案体系和管理体制，有效应对自然灾害、事故灾难、公共卫生和社会安全突发事件，提高政府应急管理和抗风险能力。

    恪尽职守，提高政府行政效能。全心全意为人民服务，做到问政于民、问需于民、问计于民，真诚倾听群众呼声，真实反映群众愿望，真情关心群众疾苦。坚持科学民主决策，一切从实际出发，自觉按规律办事，把推动发展的积极性与科学求实的态度结合起来，涉及群众利益和公共政策的重大事项要广泛听取意见，努力避免决策失误，少走弯路。努力做到“严、细、深、实、快”，大力精简会议、文件,切实改进会风、文风。推进制度创新，认真落实服务承诺制、首问负责制、限时办结制，提高办事效率。

    清正廉洁，提高政府公务员素质。以完善惩治和预防腐败体系为重点，加强反腐倡廉建设，严格落实廉政建设责任制，坚持用制度管权、管事、管人，确保权力规范运作。严格执行领导干部廉洁自律规定，勤勉尽责，秉公用权，清清白白做人，干干净净做事。树立过紧日子的观念，公务用车、会议经费、公务接待费用以及党政机关出国出境经费等支出实现零增长，严格控制新建楼堂馆所，坚决制止铺张浪费、奢靡之风。创新公务员激励约束机制，搞好公务员培训教育，开展争做“人民满意的公务员”活动，强化行政效能监察，努力建设一支政治坚定、业务精通、清正廉洁、作风优良的公务员队伍。

    各位代表！

    新的征程已经开启，新的目标催人奋进。让我们高举中国特色社会主义伟大旗帜，更加紧密地团结在以胡锦涛同志为总书记的党中央周围，以科学发展观统领经济社会发展全局，在省委、省政府和市委的坚强领导下，紧紧依靠和团结全市人民，万众一心，奋发图强，为夺取全面建设小康社会的新胜利，为建设我们美好的家园而努力奋斗，以优异的成绩迎接新中国成立60周年！
